# Efficacy, safety, and potential industry bias in using deoxycholic acid for submental fat reduction ‒ A systematic review and meta-analysis of randomized clinical trials

**DOI:** 10.1016/j.clinsp.2023.100220

**Published:** 2023-10-06

**Authors:** Gabriel Santiago Giuglio Inocêncio, Daniela Meneses-Santos, Marcelo Dias Moreira de Assis Costa, Walbert A. Vieira, Vinicius Lima de Almeida, Renata Prata Cunha Bernardes Rodrigues, Sigmar de Mello Rode, Luiz Renato Paranhos

**Affiliations:** a Postgraduate Program in Dentistry, Faculdade de Odontologia, Universidade Federal de Uberlândia, Uberlândia, MG, Brazil; b Division of Morphology, Centro de Ciências da Saúde e Biológicas, Universidade Federal de Sergipe, SE, Brazil; c Department of Restorative Dentistry, Endodontics Division, Faculdade de Odontologia de Piracicaba, Universidade Estadual de Campinas (UNICAMP), Piracicaba, SP, Brazil; d Division of Preventive and Community Dentistry, Faculdade de Odontologia, Universidade Federal de Uberlândia, Uberlândia, MG, Brazil; e Department of Dental Materials and Prothesis, Instituto de Ciência e Tecnologia, Universidade Estadual Paulista Júlio de Mesquita Filho, São José dos Campos, SP, Brazil

**Keywords:** Deoxycholic acid, Deoxycholate, Lipolysis

## Abstract

Lipolytic substance injections to reduce localized fat have been extensively used because it is a low-invasive method. This review aimed to evaluate the efficacy and safety of deoxycholic acid in submental fat reduction compared to a placebo and investigate the potential industry sponsorship bias in the results of randomized clinical trials on this topic. Ten electronic databases were extensively searched for randomized clinical trials without restriction on language and year of publication. Two reviewers extracted the data and assessed the individual risk of bias in the studies with the RoB 2.0 tool. The industry sponsorship bias was evaluated according to citations in the articles regarding industry funding/sponsorship throughout the texts. Fixed and random effects meta-analyses were performed, and the results were reported in Risk Ratio (RR) at a 95% Confidence Interval (95% CI). The initial search provided 5756 results, of which only five were included. Only two studies had a low risk of bias. All studies showed a potential industry bias. The meta-analysis showed that patients treated with deoxycholic acid had significant positive results for all efficacy outcomes and a higher risk of fibrosis, pain, erythema, numbness, swelling, edema, pruritus, nodules, headache, and paresthesia. The low to moderate certainty of evidence found allows concluding that deoxycholic acid is effective in submental fat reduction, causing well-tolerated adverse effects. However, all eligible studies showed a potential industry bias.

## Highlights

• Deoxycholic acid is effective in submental fat reduction for both clinician and patient-related outcomes.
• Well-tolerated side-effects were observed in Deoxycholic acid groups.
• All eligible randomized trials showed a potential industry bias.

## Introduction

The loss of mandibular outline in the submental region is often associated with aging, obesity,^1^ low self-esteem, and negative esthetic self-perception,^2^ which cause social and professional impacts.

The loss of the submental region outline occurs due to an accumulation of fat in the superficial (preplatysmal) and deep (postplatysmal) regions of the platysma muscle.^3^ Cryolipolysis,^4^ radiofrequency, and ultrasound^5^ stand out among the nonsurgical methods to reduce submental fat. Besides these methods, lipolytic substance injections to reduce localized fat have been extensively used^6^ because it is a low-invasive method.^7^ Deoxycholic acid is a lipolytic substance that ruptures the cell membrane of fat cells (adipocytes) and causes irreversible damage^8^ After cell death, inflammatory responses occur to remove cell debris, release intracellular fat, and recruit fibroblasts for collagen formation.^9^ However, the evidence on the effectiveness and safety of deoxycholic acid in submental fat reduction requires further explanation to indicate such treatment.

The findings of clinical trials on medications are the foundations of evidence-based practices and affect professional decision-making. However, clinical research on medical and dental procedures has been increasingly sponsored by manufacturers of drugs and devices used in the health field, either performing the studies directly or funding them completely or partially.^10^^,^^11^ Therefore, it is important to obtain information on study funding because it might be associated with biases^12^^,^^13^ that may compromise the reliability of the results.

In this context, the primary objective of the present systematic review was to evaluate the efficacy and safety of deoxycholic acid in submental fat reduction compared to a placebo and investigate the potential industry sponsorship bias in the results of randomized clinical trials.

## Material and methods

### Protocol and registration

The protocol of the present systematic review was created according to the items of the PRISMA-P (Preferred Reporting Items for Systematic Review and Meta-Analysis Protocols) report^14^ and registered in the International Prospective Register of Systematic Reviews (PROSPERO) database (http://www.crd.york.ac.uk/PROSPERO, Protocol: CRD42021234515). This systematic review was reported according to the PRISMA (Preferred Reporting Items for Systematic Reviews and Meta-Analyses)^15^ and performed according to the Joanna Briggs Institute (JBI) manual.^16^

### Study design and eligibility criteria

The systematic review was based on the research question following the PICO acronym (Population, Intervention, Comparator, and Outcome), as follows: Is using deoxycholic acid (intervention) more effective and safer for submental fat reduction (outcome) in adult patients (population) than placebo substances (comparator)?

The inclusion criteria were: 1) Only randomized clinical trials; 2) Patients older than 18 years; 3) Placebo substances as the control group; 4) The use of deoxycholic acid in any concentration as long as clearly described in the methodology; 5) Description of at least one of the success or safety criteria: Clinician-Reported Submental Fat Rating Scale (CR-SMFRS), Patient-Reported Submental Fat Rating Scale (PR-SMFRS), Subject Self-Rating Scale (SSRS), submental fat thickness reduction, and the prevalence of adverse events.

The exclusion criteria were: 1) Studies that did not consider submental fat treatments or other esthetic treatments before the procedure; 2) Studies that did not consider the body mass index of patients; 3) Studies with sample overlap (primary studies were used). Review studies, letters to the editor/editorials, personal opinions, books/book chapters, textbooks, reports, conference abstracts, and patents were also excluded.

### Sources of information, search, and selection of studies

The electronic search was performed in July 2020 and updated in January 2022 in the MedLine (via PubMed), Scopus, Cochrane Library, LILACS, SciELO, Embase, and Web of Science databases, as well as the partial search for the gray literature (OpenGrey, OpenThesis, and OATD). Additionally, a manual search in the references of the potentially eligible studies was performed to locate studies unidentified in the primary searches. Eligible studies from other systematic reviews published previously were also searched. All these steps were performed to minimize the selection bias.

Initially, the MeSH (Medical Subject Headings) terms and their synonyms were combined using the Boolean operators OR/AND to build the MedLine search strategy. Next, this strategy was adapted to the other databases, respecting their respective syntax rules (Supplementary Table 1) and using the DeCS (Health Sciences Descriptors) and Emtree (Embase Subject Headings) resources to select the search descriptors.

The studies were selected in three phases. In the first phase, the studies were identified after a bibliographical search in the databases. The results obtained were exported to the EndNote Web™ software (Thomson Reuters, Toronto, Canada), and duplicates were removed. The gray literature was exported to Microsoft Word™ 2019 (Microsoft™, Ltd, Washington, USA) to manually remove the duplicates.

Before the second phase, there was a calibration exercise in which the reviewers discussed the eligibility criteria (kappa = 0.81). In the second phase, the results were exported to the Rayyan QCRI software (Qatar Computing Research Institute, Doha, Qatar),^17^ in which titles and abstracts were analyzed according to the eligibility criteria aforementioned. Subsequently, the full texts of the preliminary eligible studies were obtained and evaluated. Two eligibility reviewers (GSGI and DMS) performed this entire process independently. Divergences were solved after consulting with a third reviewer (MDMAC), an expert in the subject.

### Data extraction

Before data extraction, to ensure consistency between the reviewers, a training exercise was performed between them (examiners GSGI and DMS), in which the data were extracted jointly from an eligible study. Any disagreement between the examiners was solved with discussions, and a third reviewer (MDMAC) was consulted to make a final decision.

Subsequently, the following data were extracted from the eligible studies: (a) Study identification (author, year, location, and type of study); (b) Sample characteristics (the number of patients, distribution by sex, average age, Body Mass Index (BMI), level of submental fat (low, moderate, or severe), and the number and interval of sessions); (c) Characteristics of the substances applied: ATX-101 and placebo substances (administration route, dose, and ATX and placebo volumes); (d) Main findings: clinical perception of submental fat before, during, and after the clinical sessions and the self-perception of submental fat before, during, and after the clinical sessions; (e) Results and conclusions (cut-off points, type of statistical analysis, and correlation index); (f) Safety outcomes: reports of % adverse events, % fibrosis, % pain, % hematoma, % swelling, % edema, % pruritus, % erythema, % numbness, % nodules, % headache, % paresthesia, and % nasopharyngitis; (g) Efficacy outcomes: Clinician-Reported Submental Fat Rating Scale (CR-SMFRS ≥1-point improvement), (CR-SMFRS ≥ 2-point improvement), Patient-Reported Submental Fat Rating Scale (PR-SMFRS ≥ 1-point improvement), (PR-SMFRS ≥2-point improvement), Subject Self-Rating Scale (SSRS), Patient-Reported Submental Fat Impact Scale (PR-SMFIS), Submental Skin Laxity Rating Scale (SLRS), and submental volume reduction ≥10%.

### Individual risk of bias

Two reviewers (WAV and GSGI) independently assessed the individual risk of bias in the eligible studies with the Risk of Bias Tool of the Cochrane Collaboration (version 2.0) (RoB2) for randomized clinical trials.^18^ This tool consists of five domains: bias from the randomization process, bias from deviations of the intended interventions, bias from missing outcome data, bias from result measurement, and bias from the selection of the reported result.

Each domain was assessed according to the algorithms proposed in the RoB2 manual. Each domain includes signaling questions that can be answered as “yes”, “probably yes”, “probably not”, “no”, or “no information”. The answers to the signaling questions show the occurrence and provide the base to judge the risk of bias at the domain level, which can be classified as follows: “high risk”, “some concerns”, or “low risk”. At the study level, the article was classified as a low risk of bias if all domains were considered “low risk”, “some concerns” if at least one domain showed some concerns, and a high risk of bias if at least one domain was considered “high risk” or several domains showed some concerns. Any disagreement between the reviewers was solved with a discussion and by consulting with a third reviewer (LRP).

### Individual risk of industry sponsorship bias

The industry bias was evaluated according to citations in the articles regarding industry funding/sponsorship throughout the texts, whether conflicts of interest or acknowledgments were stated, and whether the authors were associated with the product manufacturer. Moreover, the connections of coordinators/advisers/responsible persons for the eligible studies were evaluated, adapted from a previous study,^19^ as follows: (U) Unclear: when the authors did not report whether the manufacturer funded or sponsored the study; (√) Sponsored: when the authors reported whether there was funding or sponsorship from the manufacturer in either the conflicts of interest or acknowledgments sections of the study; (x) Not sponsored: when the authors clarified that the manufacturer did not fund or sponsor the study.

### Synthesis of results and meta-analysis

The data were summarized with the R software for Windows, version 4.1.0 (R Foundation for Statistical Computing, Vienna, Austria), aided by the meta and meta for packages. Regarding the dichotomous variables, Risk Ratio (RR) was used as the estimated effect at a 95% Confidence Interval (95% CI). Heterogeneity was determined with I^2^ statistics and classified as low (I² < 50%), moderate (I² = 50%‒75%), or high (I² > 75%). The Mantel-Haenszel fixed effect model was used when I² was ≤ 50% and the random effect model when I² > 50%.

### Assessment of the certainty of the evidence

The certainty of the evidence was assessed with the Grading of Recommendations, Assessment, Development, and Evaluation (GRADE) approach. The GRADEpro GDT software (http://gdt.guidelinedevelopment.org) summarized the results. The assessment was based on study design, risk of bias, inconsistency, indirect evidence, imprecision, and publication bias. The certainty of evidence can be classified as high, moderate, low, or very low.^20^

## Results

### Study selection

**Fig. 1:**
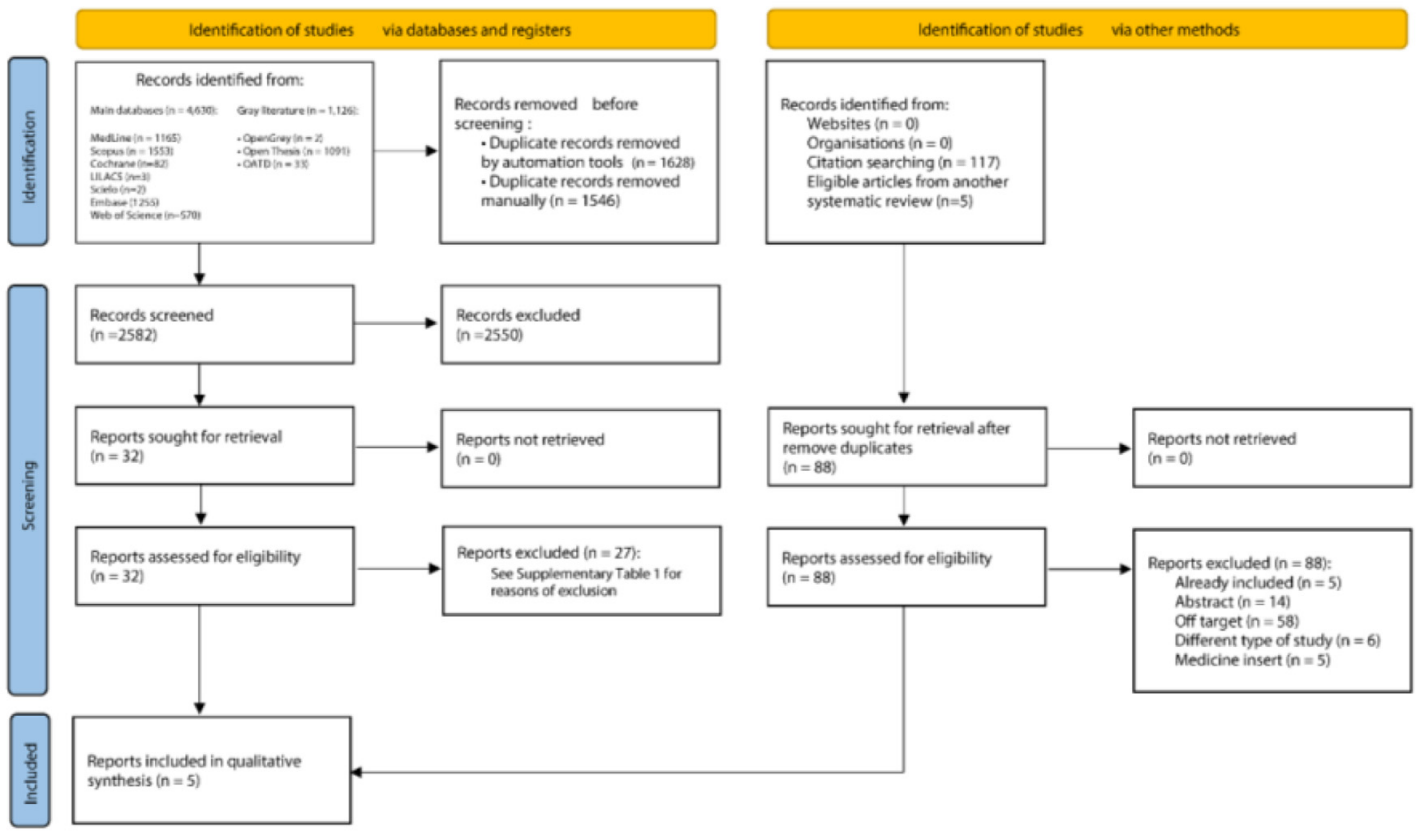
Flowchart of literature search and selection process according to the PRISMA statement.

An initial search in the scientific literature database provided 5756 results, from which 3174 duplicates were removed. The reading of titles and abstracts resulted in 2550 exclusions. After reading the full texts, 27 articles were excluded (Supplementary Table 2). Lastly, the references of the eligible studies were assessed, but no article was added. At the end of the selection, five studies^21^, ^22^, ^23^, ^24^, ^25^ were included in the qualitative and quantitative syntheses. Fig. 1 shows details of the study selection process.

### Characteristics of the eligible studies

The studies were performed between 2014 and 2019. Two studies were performed in Germany^21^^,^^22^ and three in the United States.^23^, ^24^, ^25^ A total of 1837 patients between 18 and 65 years old were assessed, of which 1744 presented levels 2 or 3 in the Clinician-Reported Submental Fat Rating Scale (CR-SMFRS), and most patients were women (*n* = 1403 ‒ 80.45%). All studies used the ATX-101 substance (KYBELLA™ in the United States and BELKYRA™ in Canada (Kythera Biopharmaceuticals Inc., Westlake Village, CA, USA, an affiliate of Allergan plc, Dublin, Ireland)). Two studies^26^^,^^27^ analyzed the efficacy of the substance and safety of doses of 1 mg/cm^2^ and 2 mg/cm^2^, and three studies^23^, ^24^, ^25^ only analyzed 2 mg/cm^2^. All studies^21^, ^22^, ^23^, ^24^, ^25^ injected a maximum volume of 10 mL per session. Two studies^21^^,^^22^ had a screening period that could include up to two visits, followed by up to four treatment sessions, and finally by two other follow-up sessions. The other three studies^23^, ^24^, ^25^ had up to six treatment sessions (Supplementary Table 3).

Among the outcomes assessed, all studies^21^, ^22^, ^23^, ^24^, ^25^ investigated Submental Fat (SMF) severity (submental convexity and amount of SMF) and the satisfaction with appearance associated with the face and chin, using the CR-SMFRS and SSRS, respectively (Table 2). Other efficacy assessment methods were the reduction of self-perceived SMF severity (PR-SMFRS) and the psychological impact of SMF (PR-SMFIS or modified DAS 24). All studies assessed submental fat thickness and skin laxity. Three studies assessed satisfaction with the treatment received.^21^^,^^24^^,^^25^ All studies^21^, ^22^, ^23^, ^24^, ^25^ took pictures and performed imaging assessments. As for safety assessment, all studies^21^, ^22^, ^23^, ^24^, ^25^ investigated Treatment-Emergent Adverse Events (TEAEs). Supplementary Tables 4 and 5 present the individual results of the eligible studies regarding efficacy and safety outcomes.

### Risk of individual bias in the studies

**Fig. 2:**
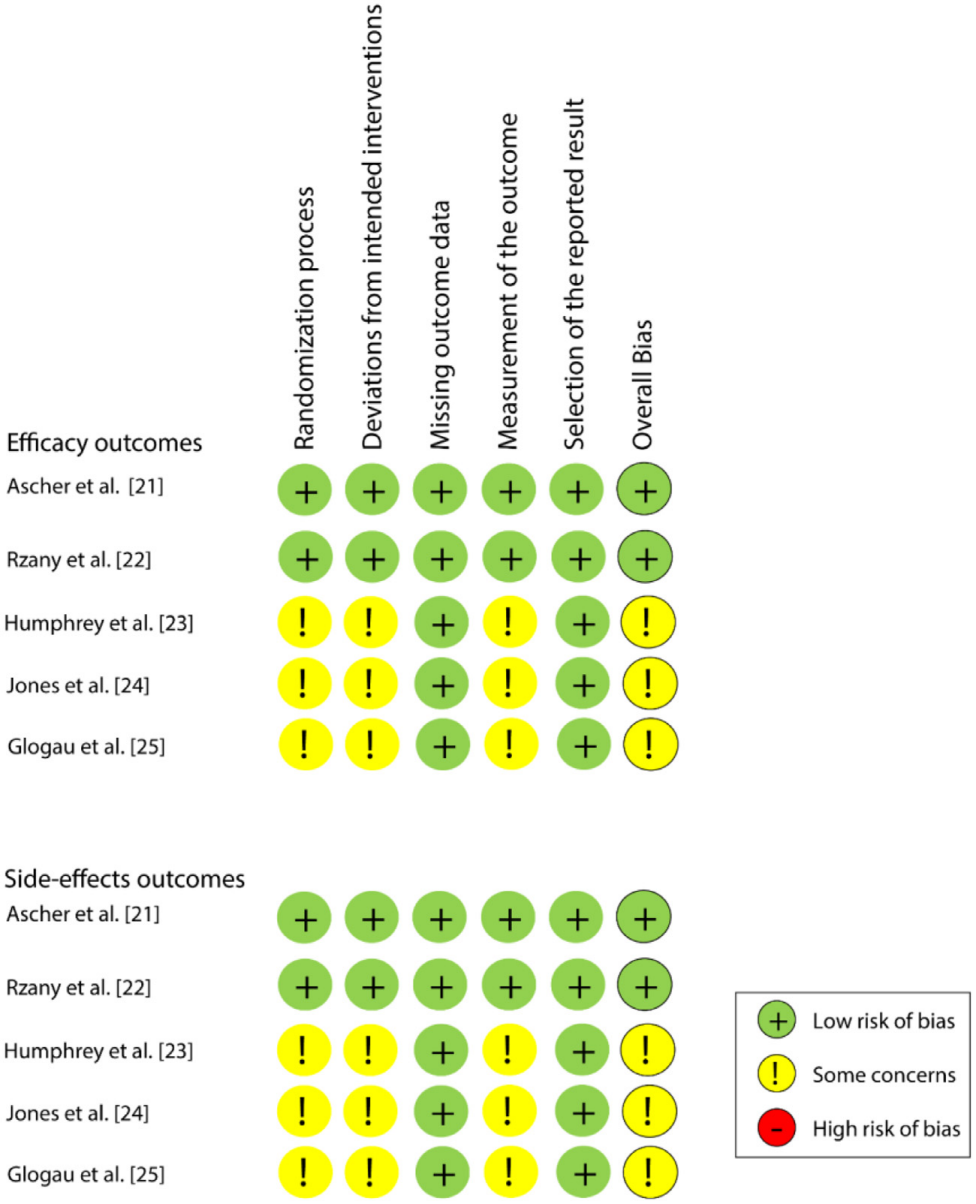
Risk of bias assessment.

Among the five studies, two^21^^,^^22^ were classified as a “low risk of bias” and the other three^23^, ^24^, ^25^ as “some concerns”, for both efficacy and safety outcomes. All studies presented a low risk of bias in the domains of “Missing outcome data” and “Selection of the reported result”. Fig. 2 shows the individual assessment of each article included.

### Risk of industry sponsorship bias

All studies reported having received company funding or sponsorship: two studies^21^^,^^22^ by Bayer HealthCare AG and KYTHERA Biopharmaceuticals Inc. and three studies^23^, ^24^, ^25^ by KYTHERA Biopharmaceuticals Inc. Moreover, all studies clarified the type of sponsorship (fees, payments, or other compensation) and the employment relationship of the authors with the companies. Regarding the last authors of each study, two studies^21^^,^^22^ were advised by author B. Havlickova and two other studies^23^^,^^24^ by author F. C. Beddingfield. The study by Glogau et al.^25^ was not advised as the others, but it shares one of the authors of the study by Jones et al.^24^ (Table 1).

**Table 1:** Industry sponsorship risk of bias assessment.

Authors	Sponsorship	Industry	Conflicts of interest	Last author (adviser)
Ascher et al.^21^	√	Bayer HealthCare and KYTHERA Biopharmaceuticals Inc.	Two authors are employees of Bayer HealthCare, and one author is a former employee and current consultant of KYTHERA Biopharmaceuticals Inc.	B. Havlickova
Rzany et al.^22^	√	Bayer HealthCare AG and KYTHERA Biopharmaceuticals Inc.	The sponsors were responsible for the study design, coordination, and compilation of the data provided by the researchers.	B. Havlickova
Humphrey et al.^23^	√	KYTHERA Biopharmaceuticals Inc.	Two authors worked as researchers for Kythera Biopharmaceuticals Inc.; three authors worked as speakers, consultants, and members of the advisory board of Kythera Biopharmaceuticals Inc.; one author was a hired employee of Kythera Biopharmaceuticals Inc. during this study; and one author was a hired employee and shareholder of Kythera Biopharmaceuticals Inc. during this trial. Also, one author was a Senior Medical Director; one author was vice-president of clinical development, biostatistics, and data management; and one author was a Medical Director of Kythera Biopharmaceuticals Inc., where they hired employees, shareholders, and stock option holders during this study. Two authors are current employees of Sienna Biopharmaceuticals Inc., Westlake Village, California, USA.	F. C. Beddingfield
Jones et al.^24^	√	KYTHERA Biopharmaceuticals Inc.	Four authors received fees, payments, or other compensation for working in this study; one author bought shares after concluding the trial; six authors were employees of Kythera Biopharmaceuticals Inc. during this study; and one author is an employee of Evidence Scientific Solutions, Philadelphia, PA, USA, and provided medical writing assistance supported by Kythera Biopharmaceuticals Inc.	F. C. Beddingfield
Glogau et al.^25^	√	KYTHERA Biopharmaceuticals Inc.	The authors received payment as researchers for this clinical trial and had previously received a subsidy, consulting fees, travel aid, and payment to develop educational material and speeches for Kythera Biopharmaceuticals Inc.	B. Bowen

The studies were classified as (U) uncertain when it was impossible to assess with certainty the sponsorship status due to missing information even after contacting the authors via e-mail, (x) not sponsored when the authors informed the study was not financially supported by companies, and (√) sponsored when the authors informed the study had some financial support from companies. The sponsorship status was defined when the authors mentioned it in the main text or the acknowledgment section of the studies, regardless of the type of sponsorship (financial support, provision of products, etc.). Both reviewers previously discussed all items to ensure consistency in their interpretation.

### Synthesis of results and meta-analysis

#### Efficacy outcomes

**Fig. 3:**
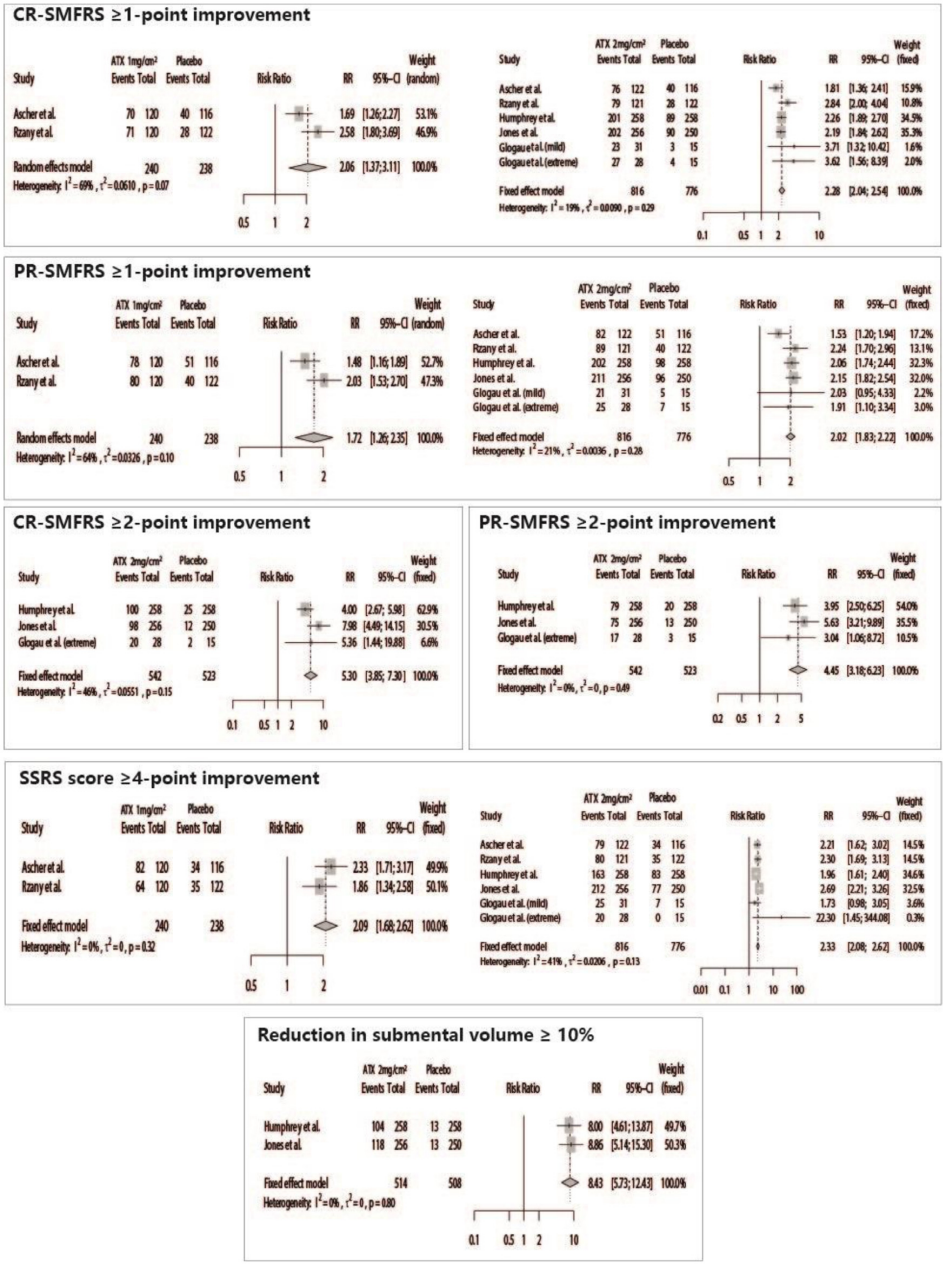
Forest plots of the meta-analyses of efficacy outcomes.

Patients treated with ATX-101 presented significant positive results for all efficacy outcomes, such as CR-SMFRS ≥1-point improvement (ATX 2 mg/cm^2^ vs. Placebo – RR = 2.28 [95% CI 2.04; 2.54]; ATX 1 mg/cm^2^ vs. Placebo – RR = 2.06 [95% CI 1.37; 3.11]), CR-SMFRS ≥ 2-point improvement (ATX 2 mg/cm^2^ vs. Placebo – RR = 5.30 [95% CI  3.85; 7.30]), PR-SMFRS ≥ 1-point improvement (ATX 2 mg/cm^2^ vs. Placebo – RR = 2.02 [95% CI 1.83; 2.22]; ATX 1 mg/cm^2^ vs. Placebo – RR = 1.72 [95% CI 1.26; 2.35]), PR-SMFRS ≥ 2-point improvement (ATX 2 mg/cm^2^ vs. Placebo – RR = 4.45 [95% CI 3.18; 6.23]), SSRS ≥4-point improvement (ATX 2 mg/cm^2^ vs. Placebo – RR = 2.33 [95% CI  2.08; 2.62]; ATX 1 mg/cm^2^ vs. Placebo – RR = 2.09 [95% CI 1.68; 2.62]), and submental volume reduction ≥ 10% (ATX 2 mg/cm^2^ vs. Placebo – RR = 8.43 [95% CI 5.73; 12.43]) (Fig. 3).

#### Safety outcomes

The analyses of safety outcomes showed that patients treated with ATX had a significantly higher risk of fibrosis (ATX 2 mg/cm^2^ vs. Placebo – RR = 9.74 [95% CI 6.08; 15.61]; ATX 1 mg/cm^2^ vs. Placebo – RR = 10.04 [95% CI 3.64; 27.68]), pain (ATX 2 mg/cm^2^ vs. Placebo – RR = 2.38 [95% CI 1.86; 3.04]; ATX 1 mg/cm^2^ vs. Placebo – RR = 3.04 [95% CI 2.46; 3.77]), hematoma (ATX 1 mg/cm^2^ vs. Placebo – RR = 1.27 [95% CI 1.07; 1.52]), erythema (ATX 2 mg/cm^2^ vs. Placebo – RR = 1.61 [95% CI 1.35; 1.93]; ATX 1 mg/cm^2^ vs. Placebo – RR = 1.80 [95% CI 1.36; 2.39]), numbness (ATX 2 mg/cm^2^ vs. Placebo – RR = 13.09 [95% CI 9.48; 18.08]; ATX 1 mg/cm^2^ vs. Placebo – RR = 21.78 [95% CI 9.04; 54.44]), swelling (ATX 2 mg/cm^2^ vs. Placebo – RR = 2.22 [95% CI 1.76; 2.79]), edema (ATX 2 mg/cm^2^ vs. Placebo – RR = 2.06 [95% CI 1.78; 2.39]), pruritus (ATX 2 mg/cm^2^ vs. Placebo – RR = 2.00 [95% CI 1.35; 2.97]), nodule (ATX 2 mg/cm^2^ vs. Placebo – RR = 5.66 [95% CI 3.16; 10.14]), headache (ATX 2 mg/cm^2^ vs. Placebo – RR = 2.07 [95% CI 1.26; 3.41]), and paresthesia (ATX 2 mg/cm^2^ vs. Placebo – RR = 3.25 [95% CI 2.04; 5.18]) (Table 2).

**Table 2:** Summary of all meta-analyses performed for safety outcomes.

Outcome	ATX concentration	No. of studies	RR (95% CI)	I² test
Edema	2 mg	3	2.06 (1.78 – 2.39)	0%
Eritema	1 mg	2	1.80 (1.36 – 2.39)	0%
	2 mg	5	1.61 (1.35 – 1.93)	0%
Fibrosis	1 mg	2	10.04 (3.64 – 27.68)	0%
	2 mg	5	9.74 (6.08 – 15.61)	0%
Headache	2 mg	3	2.07 (1.26 – 3.41)	0%
Hematoma	1 mg	2	1.27 (1.07 – 1.52)	0%
	2 mg	5	1.06 (0.98 – 1.14)	0%
Nasopharyngitis	2 mg	2	0.88 (0.57 – 1.36)	0%
Nodule	2 mg	3	5.66 (3.16 – 10.14)	20%
Numbness	1 mg	2	21.78 (9.04 – 52.44)	0%
	2 mg	5	13.09 (9.48 – 18.08)	0%
Pain	1 mg	2	3.04 (2.46 – 3.77)	0%
	2 mg	5	2.38 (1.86 – 3.04)	73%
Paresthesia	2 mg	3	3.25 (2.04 – 5.18)	50%
Pruritus	2 mg	3	2.00 (1.35 – 2.97)	0%
SLRS improvement or no change	1 mg	2	1.00 (0.94 – 1.05)	9%
	2 mg	5	1.01 (0.98 – 1.04)	0%
Swelling	2 mg	3	2.22 (1.76 – 2.79)	0%

#### Certainty of evidence

The assessment of the certainty of the evidence was divided according to the specific outcome evaluated in the analyses. Overall, the certainty of evidence varied among moderate (11 outcomes), low (16 outcomes), and very low (1 outcome). Supplementary Table 6 presents details of the assessment of each outcome for each GRADE item.

## Discussion

The present study proposed to evaluate the efficacy of deoxycholic acid in submental fat reduction, the prevalence of adverse effects, and the potential influence of industry sponsorship on the individual results of the eligible studies. The meta-analysis of the results showed that all patients treated with this substance had significantly superior results in all efficacy outcomes compared to the placebo. Moreover, the meta-analysis of safety results showed that patients who received deoxycholic acid presented a higher risk of pain, hematoma, pruritus, paresthesia, nodules, fibrosis, headache, and erythema. Regarding the potential industry sponsorship bias, all studies declared having received funding or sponsorship, and the authors had an employment relationship with the companies.

The deoxycholic acid is produced endogenously in the intestine and stored in the gallbladder.^26^ Its function consists of emulsion and solubilization of lipids to facilitate absorption by the gastrointestinal tract.^27^ The pharmaceutical industry developed the synthetic deoxycholic acid, which was previously used in association with phosphatidylcholine and amphotericin B and for treating lipomas and producing vaccines against the influenza virus.^27^^,^^28^ Later, studies were performed to evaluate the application of pure deoxycholic acid to reduce submental fat. Thus, the pure substance was named ATX-101 (commercial name Kybella™) (Kythera Biopharmaceuticals Inc.) and approved by the FDA in the United States and Canada, in 2015, becoming the first injectable drug for submental fat reduction.^29^^,^^30^

This meta-analysis found that deoxycholic acid (ATX-101), regardless of the dose (1 or 2 mg/cm²), was effective in submental fat reduction compared to the placebo. The substance presented very tolerable adverse effects, according to the eligible studies. A study by McDiarmid et al.^31^ showed that the treatment with ATX-101 reduced the CR-SMFRS score by 4.4 times and the SSRS score by 4.8 times compared to the placebo. Moreover, the study by Dover et al.^32^ showed that submental fat reduction remained for at least two to three months after the treatment with ATX-101. The authors of this study^32^ hypothesized that the lysis of the cell membrane of adipocytes associated with the tissue inflammatory response stimulates tissue remodeling and collagen synthesis in the submental region, causing a prolonged action of deoxycholic acid. Humphrey and colleagues^33^ verified that the results remained for up to three years in most patients, as well as satisfaction. It is worth noting that the assessments were subjective and not validated by previous studies.

The injected deoxycholic acid ruptures the cell membrane of adipocytes and produces an inflammatory response that removes cell debris and lipid molecules from the application site.^34^ The assessment of safety outcome measures shows that patients had a higher risk of fibrosis, pain, erythema, and numbness associated with deoxycholic acid at concentrations of 1 mg/cm^2^ and 2 mg/cm^2^ than with the placebo substances. Applying ATX may stimulate the adipocytolytic action and promote complications without well-defined causes, such as skin necrosis, nervous lesion, alopecia, and vascular events.^35^^,^^36^ An *in vitro* study by Thuangtong et al.^37^ showed that sodium deoxycholate causes lysis in the adipose tissue and the death of adipocytes. The adverse reactions usually resolve in a 28-day interval between the treatment sessions, and most complications present mild or moderate intensity.^31^

The effects of swelling, edema, pruritus, nodule, headache, and paresthesia were associated with the concentration of 2 mg/cm^2^ of deoxycholic acid. In the study by Ascher et al.,^21^ pain at the injection site, hematoma, swelling, erythema, numbness, and hardening were the most frequent adverse events. These effects may be associated with the treatment area, be temporary, and have a spontaneous regression.^21^^,^^22^ Authors affirm that, on the 28^th^ day, there is a migration of fibroblasts and remission of the inflammatory process, which is possibly why they recommend a 30-day interval between applications.^23^^,^^26^^,^^29^^,^^30^^,^^37^ All the eligible studies maintained a 28-day interval between ATX-101 injections, although 10% of patients discontinued the treatment because of the adverse effects.

The present systematic review assessed the risk of industry sponsorship bias. This bias is defined as the combination of several factors of design, data, analysis, and presentation that tend to produce rigged research results.^38^ Authors advocate those studies sponsored by the pharmaceutical industry are more prone to report results and conclusions that favor the drug instead of the placebo.^11^^,^^39^ Although 29% to 69% of clinical trials in various medical fields declare conflicts of interest,^40^ some types of study funding seem to induce positive research outcomes.^41^ Therefore, even when conflicts of interest are reported, questioning the reliability of the results is recommended. Studies assessing industry sponsorship and their results found that studies sponsored by the pharmaceutical industry are more favorable to the sponsor's product than studies with other sponsorship sources.^11^

All studies in the present review^21^, ^22^, ^23^, ^24^, ^25^ declared having received funding or sponsorship from companies and clarified the type of sponsorship and employment relationship of the authors with the companies. Bradley et al.^42^ advocate that conflict reporting or statements of interests should become more open to establishing reliability in the study objectivity.

Among the limitations of this systematic review is the low number of eligible studies that met the eligibility criteria. Among the five eligible studies, most^23^, ^24^, ^25^ showed “some concerns” in the risk of bias assessment. Although there is another recent review on the use of ATX-101 for the efficiency and safety of submental fat,^43^ the strength of the present review is assessing the industry sponsorship bias, which is important in multicenter and pharmaceutical product studies. This review used the GRADE to evaluate the certainty of the evidence, which showed outcomes ranging between moderate and low certainty of evidence.

## Conclusion

Based on a low to moderate certainty of the evidence, the meta-analyses showed positive effects regarding the efficacy of deoxycholic acid, regardless of the dose. Adverse effects with low magnitude and very tolerable were presented as safety results. All studies showed an industry sponsorship bias. Further randomized clinical trials not sponsored by the pharmaceutical industry should be encouraged to obtain independent evidence without potential conflict of interest biases.

## Authors’ contributions

All authors contributed to the conception and design of the study. G.S.G.I., M.D.M.A.C., D.M.S., and W.A.V. performed the preparation, data collection, and analysis. G.S.G.I. and V.L.A. wrote the first draft of the manuscript, and all authors commented on previous versions of the manuscript. All authors read and approved the final manuscript.

## Funding

This study was partially financed by CAPES (Coordination for the Improvement of Higher Education Personnel) ‒ Finance Code 001. The authors also appreciate the support of CNPq (National Counsel of Technological and Scientific Development ‒ Brazil) and FAPEMIG (Research Support Foundation of the State of Minas Gerais ‒ Brazil).

## Declaration of Competing Interest

The authors declare no conflicts of interest.

## Appendix Supplementary materials

Image, application 1
