# Supplementary material for: Efficacy, safety, and potential industry bias in using deoxycholic acid for submental fat reduction ‒ A systematic review and meta-analysis of randomized clinical trials
Source: Clinics (Sao Paulo). 2023 Oct 6;78:100220. doi: 10.1016/j.clinsp.2023.100220 (PMC10570630; doi:10.1016/j.clinsp.2023.100220)
Supplement: Supplementary file 1 [file mmc1.docx]

CLINICS-D-22-00616_Supplementary Material

**Supplementary Table 1** Database search strategy.

| **Database** | **Search Strategy** |
| --- | --- |
| PubMed http://www.ncbi.nlm.nih.gov/pubmed | ((“Deoxycholic Acid” OR “Deoxycholate acid” OR “Desoxycholic Acid” OR “Dihydroxycholanoic Acid” OR “ATX-101” OR “Deoxycholate”) AND (“Adipocytolysis” OR “Submental fat” OR “Reduction” OR “Lipolysis” OR “Lipectomy” OR “Lipoplasty”)) |
|  |  |
| Scopus http://www.scopus.com | (((“Deoxycholic Acid” OR “Deoxycholate acid” OR “Desoxycholic Acid” OR “Dihydroxycholanoic Acid” OR “ATX-101” OR “Deoxycholate”) AND (“Adipocytolysis” OR “Submental fat” OR “Reduction” OR “Lipolysis” OR “Lipectomy” OR “Lipoplasty”))) |
|  |  |
| Cochrane Library https://www.cochranelibrary.com | ((“Deoxycholic Acid” OR “Deoxycholate acid” OR “Desoxycholic Acid” OR “Dihydroxycholanoic Acid” OR “ATX-101” OR “Deoxycholate”) AND (“Adipocytolysis” OR “Submental fat” OR “Reduction” OR “Lipolysis” OR “Lipectomy” OR “Lipoplasty”)) |
| LILACS http://lilacs.bvsalud.org/ | (“deoxycholic acid” OR “deoxycholate acid” OR “desoxycholic acid” OR “dihydroxycholanoic acid” OR “atx-101” OR “deoxycholate”) AND (“adipocytolysis” OR “submental fat” OR “reduction” OR “lipolysis” OR “lipectomy” OR “lipoplasty”) AND (db:("LILACS")) |
|  |  |
| SciELO http://www.scielo.org/ | Deoxycholic acid AND adipocytolysis |
|  | Deoxycholic acid AND submental fat |
|  | Deoxycholic acid AND reduction |
|  | Deoxycholic acid AND lipolysis |
|  | Deoxycholic acid AND lipectomy |
|  | Deoxycholic acid AND lipoplasty |
|  | Deoxycholate acid AND adipocytolysis |
|  | Deoxycholate acid AND submental fat |
|  | Deoxycholate acid AND reduction |
|  | Deoxycholate acid AND lipolysis |
|  | Deoxycholate acid AND lipectomy |
|  | Deoxycholate acid AND lipoplasty |
|  | Desoxycholic acid AND adipocytolysis |
|  | Desoxycholic acid AND submental fat |
|  | Desoxycholic acid AND reduction |
|  | Desoxycholic acid AND lipolysis |
|  | Desoxycholic acid AND lipectomy |
|  | Desoxycholic acid AND lipoplasty |
|  | Dihydroxycholanoic acid AND adipocytolysis |
|  | Dihydroxycholanoic acid AND submental fat |
|  | Dihydroxycholanoic acid AND reduction |
|  | Dihydroxycholanoic acid AND lipolysis |
|  | Dihydroxycholanoic acid AND lipectomy |
|  | Dihydroxycholanoic acid AND lipoplasty |
|  | ATX-101 AND adipocytolysis |
|  | ATX-101 AND submental fat |
|  | ATX-101 AND reduction |
|  | ATX-101 AND lipolysis |
|  | ATX-101 AND lipectomy |
|  | ATX-101 AND lipoplasty |
|  | Deoxycholate AND adipocytolysis |
|  | Deoxycholate AND submental fat |
|  | Deoxycholate AND reduction |
|  | Deoxycholate AND lipolysis |
|  | Deoxycholate AND lipectomy |
|  | Deoxycholate AND lipoplasty |
|  |  |
| Embase http://www.embase.com | ('deoxycholic acid'/exp OR 'deoxycholic acid' OR 'deoxycholate acid' OR 'desoxycholic acid'/exp OR 'desoxycholic acid' OR 'dihydroxycholanoic acid'/exp OR 'dihydroxycholanoic acid' OR 'atx-101'/exp OR 'atx-101' OR 'deoxycholate'/exp OR 'deoxycholate') AND ('adipocytolysis' OR 'submental fat'/exp OR 'submental fat' OR 'reduction'/exp OR 'reduction' OR 'lipolysis'/exp OR 'lipolysis' OR 'lipectomy'/exp OR 'lipectomy' OR 'lipoplasty'/exp OR 'lipoplasty') |
| Web Of Science http://apps.webofknowledge.com/ | (((“Deoxycholic Acid” OR “Deoxycholate acid” OR “Desoxycholic Acid” OR “Dihydroxycholanoic Acid” OR “ATX-101” OR “Deoxycholate”) AND (“Adipocytolysis” OR “Submental fat” OR “Reduction” OR “Lipolysis” OR “Lipectomy” OR “Lipoplasty”))) |
|  |  |
| OpenGrey http://www.opengrey.eu/ | ((“Deoxycholic Acid” OR “Deoxycholate acid” OR “Desoxycholic Acid” OR “Dihydroxycholanoic Acid” OR “ATX-101” OR “Deoxycholate”) AND (“Adipocytolysis” OR “Submental fat” OR “Reduction” OR “Lipolysis” OR “Lipectomy” OR “Lipoplasty”)) |
|  |  |
| OpenThesis http://www.openthesis.org/ | ("deoxycholic acid" OR "deoxycholate acid" OR "desoxycholic acid" OR "dihydroxycholanoic acid" OR "atx-101" OR "deoxycholate") AND ("adipocytolysis" OR "submental fat" OR "reduction" OR "lipolysis" OR "lipectomy" OR "lipoplasty") |
|  |  |
| Open Access Theses and Dissertations (OATD) https://oatd.org/ | (("deoxycholic acid" OR "deoxycholate acid" OR "desoxycholic acid" OR "dihydroxycholanoic acid" OR "atx-101" OR "deoxycholate") AND ("adipocytolysis" OR "submental fat" OR "reduction" OR "lipolysis" OR "lipectomy" OR "lipoplasty")) |

**Supplementary Table 2** Studies excluded after reading the full texts, and the reasons for exclusion (n = 27).

|  | **Study excluded** | **Reason for exclusion** |
| --- | --- | --- |
| 1 | Rotunda et al., 2009 | Associated with another substance |
| 2 | Ogden & Griffiths, 2011 | Abstract |
| 3 | Pinto et al., 2014 | The study design differs from the objective |
| 4 | McDiarmid et al., 2014 | The study design differs from the objective |
| 5 | Rauso & Salti, 2015 | The study design differs from the objective |
| 6 | Walker & Lee, 2015 | The study design differs from the objective |
| 7 | Beer et al., 2015 | Abstract |
| 8 | Fabi et al., 2016 | Abstract |
| 9 | Jones et al., 2016 | Technique description |
| 10 | Beer et al., 2016 | Abstract |
| 11 | Humphrey, 2016 | Review |
| 12 | Shridharani, 2017 | The study design differs from the objective |
| 13 | Shridharani & Behr, 2017 | Retrospective review |
| 14 | Grow et al., 2018 | The study design differs from the objective |
| 15 | Rauso et al., 2018 | Letter to the editor |
| 16 | Dover et al., 2018 | Letter to the editor |
| 17 | Beer et al., 2019 | The study design differs from the objective (no control group) |
| 18 | Behr et al., 2019 | The study design differs from the objective |
| 19 | Rauso, 2019 | The study design differs from the objective |
| 20 | Shridharani, 2019 | The study design differs from the objective |
| 21 | Shridharani & Chandawarkar, 2019 | Retrospective review |
| 22 | Shome et al., 2019 | The study design differs from the objective |
| 23 | Zarbafian et al., 2019 | The study design differs from the objective |
| 24 | Palm et al., 2019 | Abstract |
| 25 | Nathan et al., 2020 | Abstract |
| 26 | Cunha et al., 2021 | Review |
| 27 | Goodman et al., 2021 | The study design differs from the objective |

**References**

1. Rotunda AM. Injectable treatments for adipose tissue: terminology, mechanism, and tissue interaction. Lasers Surg Med. 2009;41(10):714-20.

2. Ogden S, Griffiths T. A novel injectable drug for the reduction of localized fat. Br J Dermatol. 2011;165(1):98-9.

3. Pinto H, Hernandez C, Turra C, Manzano M, Salvador L, Tejero P. Evaluation of a new adipocytolytic solution: adverse effects and their relationship with the number of vials injected. J Drugs Dermatol JDD. 2014;13(12):1451-5.

4. McDiarmid J, Ruiz JB, Lee D, Lippert S, Hartisch C, Havlickova B. Results from a pooled analysis of two European, randomized, placebo-controlled, phase 3 studies of ATX-101 for the pharmacologic reduction of excess submental fat. Aesthetic Plast Surg. 2014;38(5):849-60.

5. Salti G, Rauso R. Facial Rejuvenation with Fillers: The Dual Plane Technique. J Cutan Aesthetic Surg. 2015;8(3):127-33.

6. Walker P, Lee D. A phase 1 pharmacokinetic study of ATX-101: serum lipids and adipokines following synthetic deoxycholic acid injections. J Cosmet Dermatol. 2015;14(1):33-9.

7. Beer KR, Donofrio L, Gross TM, et al. Clinically meaningful reduction in submental fat during and after treatment with ATX101 in the US/CAN phase 3 trials, REFINE-1, and REFINE-2. Presented at 73^rd^ Annual Meeting of the American Academy of Dermatology, San Francisco, CA, March 20–24, 2015.

8. Fabi S, Draelos Z; Dayan HS, Gross TM, Lizzul PF, Beddingfield FC 3rd, et al. Changes in Skin Laxity Among Responders to ATX-101 (Deoxycholic Acid Injection) in the REFINE Trials. J Clin Aesthet Dermatol. 2016;9(5 Suppl 1): S6-S7.

9. Jones DH, Kenkel JM, Fagien S, Glaser DA, Monheit GD, Stauffer K, et al. Proper Technique for Administration of ATX-101 (Deoxycholic Acid Injection): Insights from an Injection Practicum and Roundtable Discussion. Dermatol Surg Off Publ Am Soc Dermatol Surg Al. 2016;42 Suppl 1:S275-81.

10. Beer KR, Weinkle SH, Cox SE C, Rubin MG, Adelglass JM, Lizzul PF, et al. Safety and Efficacy of ATX-101 (Deoxycholic Acid Injection) for Reduction of Submental Fat: Results from a Multicenter, Open - Label Phase 3b Trial. J Clin Aesthet Dermatol. 2016;9(5 Suppl 1): S7.

11. Humphrey S, Beleznay K, Beleznay JDA. Sodium Deoxycholate for Submental Contouring. Skin Ther Lett. 2016;21(5):1-4.

12. Shridharani SM. Early Experience in 100 Consecutive Patients With Injection Adipocytolysis for Neck Contouring With ATX-101 (Deoxycholic Acid). Dermatol Surg. 2017;43(7):950-8.

13. Shridharani SM, Behr KL. ATX-101 (Deoxycholic Acid Injection) Treatment in Men: Insights From Our Clinical Experience. Dermatol Surg Off Publ Am Soc Dermatol Surg. 2017;43 Suppl 2:S225-30.

14. Grow JN, Holding J, Korentager R. Assessing the Efficacy of Deoxycholic Acid for the Treatment of Submental Fat: A Three-Dimensional Study. Aesthet Surg J. 2019;39(12):1400-11.

15. Rauso R, Tartaro G, Rugge L, Chirico F, Zerbinati N. Remodeling the neck and the lower jaw with deoxycholate injections. J Biol Regul Homeost Agents. 2018;32(5):1279-83.

16. Dover JS, Shridharani SM, Bloom JD, Somogyi C, Gallagher CJ. Reduction of Submental Fat Continues Beyond 28 Days After ATX-101 Treatment: Results From a Post hoc Analysis. Dermatol Surg. 2018;44(11):1477-9.

17. Beer K, Weinkle SH, Cox SE, Rubin MG, Shamban A, Somogyif C. ATX-101 (Deoxycholic Acid Injection) for Reduction of Submental Fat: Results From a 12-Month Open-Label Study. J Drugs Dermatol JDD. 2019;18(9):870-7.

18. Behr K, Kavali CM, Munavalli G, Teller CF, Yoelin S, Breshears L, et al. ATX-101 (Deoxycholic Acid Injection) Leads to Clinically Meaningful Improvement in Submental Fat: Final Data From CONTOUR. Dermatol Surg Off Publ Am Soc Dermatol Surg. 2020;46(5):639-45.

19. Rauso R. Deoxycholate (ATX-101) Mixed with Lidocaine to Minimize Pain/Discomfort in Nonsurgical Treatment of Submental Fullness Appearance. J Cutan Aesthetic Surg. 2018;11(4):229-33.

20. Shridharani SM. Real-World Experience With 100 Consecutive Patients Undergoing Neck Contouring With ATX-101 (Deoxycholic Acid): An Updated Report With A 2-Year Analysis. Dermatol Surg. 2019;45(10):1285-93.

21. Shridharani SM, Chandawarkar AA. Novel Expanded Safe Zone for Reduction of Submental Fullness with ATX-101 Injection. Plast Reconstr Surg. 2019;144(6):995e-1001e.

22. Shome D, Khare S, Kapoor R. The Use of Deoxycholic Acid for the Clinical Reduction of Excess Submental Fat in Indian Patients. J Drugs Dermatol JDD. 2019;18(3):266-72.

23. Zarbafian M, Karavan M, Greene R, Fabi SG. Efficacy and safety of ATX-101 as a treatment for submental fullness: A retrospective analysis of two aesthetic practices. J Cosmet Dermatol. 2020;19(6):1328-32.

24. Palm MD, Schlessinger J, Callender VD, Fagien S, Beer K, Magante S, et al. Final Data from the Condition of Submental Fullness and Treatment Outcomes Registry (CONTOUR). J Drugs Dermatol JDD. 2019;18(1):40-8.

25. Nathan NR, Pollock SE, Kourosh AS. A novel protocol for the use of deoxycholic acid in body contouring mitigating pain and inflammation while maintaining efficacy: Piloted on different body sites. Int J Womens Dermatol. 2020;6(3):233-4.

26. Cunha KS, Lima F, Cardoso RM. Efficacy and safety of injectable deoxycholic acid for submental fat reduction: a systematic review and meta-analysis of randomized controlled trials. Expert Rev Clin Pharmacol. 2021;14(3):383-97.

27. Goodman GJ, Spelman LJ, Lowe N, Bowen B. Randomized, Placebo-Controlled Phase 1/2 Study to Determine the Appropriate ATX-101 Concentration for Reduction of Submental Fat. Dermatol Surg Off Publ Am Soc Dermatol Surg. 2021;47(8):1065-70.

**Supplementary Table 3** Main characteristics of the eligible studies.

| **Authorship, year of publication, and country of origin** | **Ascher et al., 2014 Germany** | **Rzany et al., 2014 Germany** | **Humphrey et al., 2016 USA** | **Jones et al., 2016 USA** | **Glogau et al., 2019 USA** |
| --- | --- | --- | --- | --- | --- |
| Sample size and sex | 100 (♂) 260 (♀) | 85 (♂) 277 (♀) | 71 (♂) 445 (♀) | 85 (♂) 421 (♀) | 15 (♂) 78 (♀) |
| Age group | 18‒65 years | 18‒65 years | 18‒65 years | 18‒65 years | 18‒65 years |
| Age (average) | 46.0 | 46.4 | 47.9 | 49.4 | 50.0 |
| Body mass index (mean) | 26.3 kg/m^2^ | 25.7 kg/m^2^ | 29.3 kg/m^2^ | 29 kg/m^2^ | 28.4 kg/m^2^ |
|  |  |  |  |  |  |
| Number of sessions | Up to 4 sessions with a 28-day interval between each session | Up to 4 sessions with a 28-day interval between each session | Up to 6 treatments with a 28-day interval between each session | Up to 6 treatments with a 28-day interval between each session | Up to 6 treatments with a 28-day interval between each session |
| CR-SMFRS | Level 2 or 3 | Level 2 or 3 | Level 2 or 3 | Level 2 or 3 | Levels 1 to 4 |
| Administration route | Subcutaneous injections in preplatysmal fat. | Subcutaneous injections in preplatysmal fat. | Subcutaneous injections in preplatysmal fat. | Subcutaneous injections in preplatysmal fat. | Subcutaneous injections in preplatysmal fat. |
| ATX-101 dose | 1 mg/cm^2^ and 2 mg/cm^2^ | 1 mg/cm^2^ and 2 mg/cm^2^ | 2 mg/cm^2^ | 2 mg/cm^2^ | 2 mg/cm^2^ |
| Mean total ATX-101 volume | 16.96 ± 7.87 mL – 1 mg/cm^2^ | ‒ | 25.6 ± 14.6 mL | 25.0 ± 13.4 mL | 22.6 ± 12.9 mL |
|  | 15.02 ± 8.24 mL – 2 mg/cm^2^ |  |  |  |  |
| Mean total placebo substance volume | 19.16 ± 8.03 mL | ‒ | 32.3 ± 15.1 mL | 33.3 ± 12.9 mL | 27.8 ± 13.7 mL |
| Application | 0.2 mL per ATX-101 injection | 0.2 mL per ATX-101 injection | 0.2 mL per ATX-101 injection | 0.2 mL per ATX-101 injection | ‒ |
| Placebo substance | Sodium phosphate and sodium chloride in water for injection | Sodium phosphate and sodium chloride in water for injection | Buffered saline solution with phosphate preserved in 0.9% benzyl alcohol | ‒ | ‒ |
| Efficacy result (primary) | Treatment respondents regarding the CR-SMFRS score of 5 points (≥1-point improvement of the baseline in SMF reduction) | Treatment respondents regarding the CR-SMFRS score of 5 points (≥1-point improvement of the baseline in SMF reduction) | Treatment respondents regarding the CR-SMFRS score of 5 points (≥1- and ≥2-point improvement of the baseline in SMF reduction) | Treatment respondents regarding the CR-SMFRS score of 5 points (≥1- and ≥2-point improvement of the baseline in SMF reduction) | Treatment respondents regarding the CR-SMFRS score of 5 points (≥1- and ≥2-point improvement of the baseline in SMF reduction) |
|  | SSRS score of 7 points (patients satisfied with their appearance in association with chin and face, SSRS ≥4) | SSRS score of 7 points (patients satisfied with their appearance in association with chin and face, SSRS ≥4) | Proportion of patients with SMF reduction of ≥1 and ≥2 points in the Patient-Reported Submental Fat Rating Scale (PR-SMFRS) | Proportion of patients with SMF reduction of ≥1 and ≥2 points in the Patient-Reported Submental Fat Rating Scale (PR-SMFRS) | Proportion of patients with SMF reduction of ≥1 and ≥2 points in the Patient-Reported Submental Fat Rating Scale (PR-SMFRS) |
|  | SMF reduction of ≥1 point in the Patient-Reported Submental Fat Rating Scale (PR-SMFRS) | SMF reduction of ≥1 point in the Patient-Reported Submental Fat Rating Scale (PR-SMFRS) | SSRS score of 7 points (patients satisfied with their appearance in association with chin and face, SSRS ≥4) | SSRS score of 7 points (patients satisfied with their appearance in association with chin and face, SSRS ≥4) | SSRS score of 7 points (patients satisfied with their appearance in association with chin and face, SSRS ≥4) |
|  | Patient responses to the Modified Derriford Appearance Scale 24 (modified DAS 24) questionnaire | Effect of the treatment on the psychological impact of SMF using the Patient-Reported Submental Fat Impact Scale (PR-SMFIS) | Effect of the treatment on the psychological impact of SMF using the Patient-Reported Submental Fat Impact Scale (PR-SMFIS) | Effect of the treatment on the psychological impact of SMF using the Patient-Reported Submental Fat Impact Scale (PR-SMFIS) | Effect of the treatment on the psychological impact of SMF using the Patient-Reported Submental Fat Impact Scale (PR-SMFIS) |
|  |  |  | SMF thickness | SMF thickness | SMF thickness |
|  | Effect of clinician-assessed treatment on skin laxity (Skin Laxity Rating Scale [SLRS]) | Patient responses to the Modified Derriford Appearance Scale 24 (modified DAS 24) questionnaire | Proportion of patients satisfied with the fat under their chins, the outline between the chin and the neck, and treatment | Proportion of patients satisfied with the fat under their chins, the outline between the chin and the neck, and treatment | Subject perception of SMF reduction |
|  | Proportion of patients satisfied with the fat under their chins, the outline between the chin and the neck, and treatment (Subject Global Questions) | Effect of clinician-assessed treatment on skin laxity (Skin Laxity Rating Scale [SLRS]) | Effect of clinician-assessed treatment on skin laxity (Skin Laxity Rating Scale [SLRS]) | Effect of clinician-assessed treatment on skin laxity (Skin Laxity Rating Scale [SLRS]) | Attractiveness self-ratings of the chin/neck area |
|  |  | SMF thickness | Submental volume (via magnetic resonance imaging) | Submental volume (via magnetic resonance imaging) |  |
|  |  | Proportion of patients satisfied with the fat under their chins, the outline between the chin and the neck, and treatment (Subject Global Questions) |  |  |  |
| Safety result measures | Emergent adverse effects | Emergent adverse effects | Emergent adverse effects | Emergent adverse effects | Emergent adverse effects |

^a^ Distribution by non-specified sex.

♂, Male; ♀, Female; SMF, Submental Fat; CR-SMFRS, Clinician-Reported SMF Rating Scale; SSRS, Subject Self-Rating Scale; PR-SMFRS, Patient-Reported SMF Rating Scale; PR-SMFIS, Patient-Reported SMF Impact Scale; Modified DAS 24, Modified Derriford Appearance Scale 24; SGQ, Subject Global Questions; SLRS, Skin Laxity Rating Scale; EAs, Adverse Events; SLRS, Submental Skin Laxity Rating Scale; +, Scales used in the primary assessment.

**Supplementary Table 4** Main individual results of efficacy outcomes, in percentage.

| **Study** | **Experimental groups** | **CR-SMFRS ≥1-point improvement** | **CR-SMFRS ≥2-point improvement** | **PR-SMFRS ≥1-point improvement** | **PR-SMFRS ≥2-point improvement** | **SSRS ≥4-point improvement** | **PR-SMFIS, points** | **SMF thickness reduction** | **Submental volume reduction ≥10%** |
| --- | --- | --- | --- | --- | --- | --- | --- | --- | --- |
| Ascher et al., 2014 | ATX1 | 58.3 | n.a. | 64.9 | n.a. | 68.3 | n.r. | n.r. | n.a. |
|  | ATX2 | 62.3 | n.a. | 67.3 | n.a. | 64.8 | n.r. | n.r. | n.a. |
|  | PLA | 34.5 | n.a. | 44.1 | n.a. | 29.3 | n.r. | n.r. | n.a. |
| Rzany et al., 2014 | ATX1 | 59.2 | n.a. | 67.0 | n.a. | 53.3 | n.r. | -3.8 mm | n.a. |
|  | ATX2 | 65.3 | n.a. | 73.6 | n.a. | 66.1 | n.r. | -4.2 mm | n.a. |
|  | PLA | 23.0 | n.a. | 32.4 | n.a. | 28.7 | n.r. | -1.7 mm | n.a. |
| Humphrey et al., 2016 | ATX2 | 77.9 | 38.7 | 78.4 | 30.8 | 63.1 | -3.7^c^ | -17.8 ± 1.3^a^ | 40.2 |
|  | PLA | 34.5 | 9.9 | 37.8 | 7.9 | 32.2 | -1.5^c^ | -8.4 ± 1.3^a^ | 5.2 |
| Jones et al., 2016 | ATX2 | 79.1 | 38.3 | 82.3 | 29.3 | 82.8 | -3.7^c^ | -21.9 ± 1.3^a^ | 46.3 |
|  | PLA | 36.2 | 5.0 | 38.5 | 5.4 | 31.0 | -1.2^c^ | -6.2 ± 1.3^a^ | 5.3 |
| Glogau et al., 2019 | ATX2-M | 74.2 | n.a. | 67.7 | n.a. | 80.0 | -4.4^c^ | -3.5 ± 0.5^b^ | n.a. |
|  | PLA-M | 20.0 | n.a. | 33.3 | n.a. | 46.7 | -0.7^c^ | -1.1 ± 0.7^b^ | n.a. |
|  | ATX2-E | 96.0 | 71.4 | 89.3 | 60.7 | 71.4 | -4.3^c^ | -6.9 ± 0.6^b^ | n.a. |
|  | PLA-E | 26.7 | 13.3 | 46.7 | 20.0 | 0.0 | -0.7^c^ | -1.3 ± 0.9^b^ | n.a. |

n.a., Not Assessed; n.r., Impossible to Extract.

^a^ Data in reduction percentage (mean).

^b^ Data in reduction percentage (median).

^c^ Data in reduction points.

**Supplementary Table 5** Main individual safety results, in percentage.

| **Study** | **Experimental groups** | **Laxity assessment** | **Withdrawals due to AE** | **TEAEs** | **Fibrosis** | **Pain** | **Hematoma** | **Swelling** | **Edema** | **Pruritus** | **Erythema** | **Numbness** | **Nodule** | **Headache** | **Paresthesia** | **Nasopharyngitis** |
| --- | --- | --- | --- | --- | --- | --- | --- | --- | --- | --- | --- | --- | --- | --- | --- | --- |
| Ascher et al., 2014 | ATX1 | 91.0 | 5.9 | 99.2 | 15.3 | 90.1 | 60.2 | n.r. | n.r. | n.r. | 42.4 | 44.1 | n.r. | n.r. | n.r. | n.r. |
|  | ATX2 | 92.8 | 11.5 | 99.2 | 19.8 | 90.2 | 53.3 | n.r. | n.r. | n.r. | 42.6 | 52.5 | n.r. | n.r. | n.r. | n.r. |
|  | PLA | 94.1 | 0.9 | 78.9 | 0.9 | 29.8 | 50.0 | n.r. | n.r. | n.r. | 21.9 | 1.8 | n.r. | n.r. | n.r. | n.r. |
| Rzany et al., 2014 | ATX1 | 91.4 | 7.5 | 90.8 | 18.5 | 77.3 | 55.0 | n.r. | n.r. | n.r. | 38.7 | 47.9 | n.r. | n.r. | n.r. | n.r. |
|  | ATX2 | 88.2 | 8.2 | 95.0 | 26.4 | 80.2 | 53.7 | n.r. | n.r. | n.r. | 37.2 | 51.2 | n.r. | n.r. | n.r. | n.r. |
|  | PLA | 89.1 | 0.8 | 50.8 | 2.5 | 25.4 | 41.0 | n.r. | n.r. | n.r. | 23.0 | 2.5 | n.r. | n.r. | n.r. | n.r. |
| Humphrey et al., 2016 | ATX2 | 94.1 | 6.5 | 97.7 | 28.3 | 73.6 | 72.9 | 29.1 | 67.8 | 16.3 | 35.3 | 65.5 | 14.3 | 8.9 | 14.7 | 4.7 |
|  | PLA | 94.8 | 1.1 | 92.2 | 3.5 | 39.1 | 72.7 | 15.6 | 36.3 | 8.2 | 25.4 | 7.0 | 4.3 | 3.1 | 4.3 | 5.1 |
| Jones et al., 2016 | ATX2 | 92.7 | 7.4 | 96.9 | 18.3 | 65.4 | 70.0 | 37.4 | 52.9 | 8.6 | 17.9 | 66.9 | 12.5 | 7.4 | 12.8 | 8.9 |
|  | PLA | 87.6 | 1.2 | 86.4 | 1.6 | 23.4 | 67.3 | 15.7 | 21.8 | 3.6 | 10.1 | 4.4 | 0.8 | 4.4 | 3.2 | 10.5 |
| Glogau et al., 2019 | ATX2-M | 100 | 10.0 | 96.8 | 12.9 | 74.2 | 48.4 | 41.9 | 41.9 | 12.9 | 6.5 | 48.4 | 12.9 | 6.5 | 6.5 | n.r. |
|  | PLA-M | 100 | 0.0 | 93.8 | 0.0 | 50.0 | 50.0 | 12.5 | 25.0 | 6.3 | 0.0 | 6.3 | 0.0 | 6.3 | 12.5 | n.r. |
|  | ATX2-E | 100 | 16.7 | 96.6 | 10.0 | 53.6 | 60.0 | 46.7 | 46.7 | 10.0 | 10.0 | 53.3 | 13.3 | 10.0 | 0.0 | n.r. |
|  | PLA-E | 93.3 | 0.0 | 81.3 | 6.3 | 31.3 | 56.3 | 12.5 | 18.8 | 12.5 | 6.3 | 6.3 | 0.0 | 6.3 | 0.0 | n.r. |

n.a., Not Assessed; n.r., Impossible to Extract

^a^ Data in reduction percentage (mean); ^b^ Data in reduction percentage (median).

**Supplementary Table 6** Classification of the GRADE recommendations.

| **Certainty assessment** | | | | | | | **Number of patients** | |  | **Certainty** |
| --- | --- | --- | --- | --- | --- | --- | --- | --- | --- | --- |
| **Number of studies** | **Study design** | **Risk of bias** | **Inconsistency** | **Indirectness** | **Imprecision** | **Other considerations** | **Intervention** | **Control** | **Relative effect (95% CI)** |  |
| CR-SMFRS ≥ 1-point improvement (ATX 1 mg/cm^2^ vs. Placebo) | | | | | | | | | | |
| 2 | RCT | Serious^a^ | Not serious | Not serious | Serious^b^ | None^c^ | 141/240 (58.7%) | 68/238 (28.6%) | OR 2.06 (1.37 to 3.11) | ⨁⨁◯◯ LOW |
| CR-SMFRS ≥ 1-point improvement (ATX 2 mg/cm^2^ vs. Placebo) | | | | | | | | | | |
| 5 | RCT | Serious^a^ | Not serious | Not serious | Not serious | None^c^ | 608/816 (74.5%) | 254/776 (32.7%) | OR 2.28 (2.04 to 2.54) | ⨁⨁⨁◯ MODERATE |
| CR-SMFRS ≥ 2-point improvement (ATX 2 mg/cm^2^ vs. Placebo) | | | | | | | | | | |
| 3 | RCT | Serious^a^ | Not serious | Not serious | Serious^b^ | None^c^ | 218/542 (40.2%) | 39/523 (7.5%) | OR 5.30 (3.85 to 7.30) | ⨁⨁◯◯ LOW |
| PR-SMFRS ≥ 1-point improvement (ATX 1 mg/cm^2^ vs. Placebo) | | | | | | | | | | |
| 2 | RCT | Serious^a^ | Not serious | Not serious | Serious^b^ | None^c^ | 158/240 (65.3%) | 91/238 (38.2%) | OR 1.72 (1.26 to 2.35) | ⨁⨁◯◯ LOW |
| PR-SMFRS ≥ 1-point improvement (ATX 2 mg/cm^2^ vs. Placebo) | | | | | | | | | | |
| 5 | RCT | Serious^a^ | Not serious | Not serious | Not serious | None^c^ | 630/816 (77.2%) | 297/776 (38.3%) | OR 2.02 (1.83 to 2.22) | ⨁⨁⨁◯ MODERATE |
| PR-SMFRS ≥ 2-point improvement (ATX 2 mg/cm^2^ vs. Placebo) | | | | | | | | | | |
| 3 | RCT | Serious^a^ | Not serious | Not serious | Serious^b^ | None^c^ | 171/542 (31.5%) | 36/523 (6.9%) | OR 4.45 (3.18 to 6.23) | ⨁⨁◯◯ LOW |
| SSRS ≥4-point improvement (ATX 1 mg/cm^2^ vs. Placebo) | | | | | | | | | | |
| 2 | RCT | Serious^a^ | Not serious | Not serious | Serious^b^ | None^c^ | 146/240 (60.8%) | 69/238 (30.0%) | OR 2.09 (1.68 to 2.62) | ⨁⨁◯◯ LOW |
| SSRS ≥4-point improvement (ATX 2 mg/cm^2^ vs. Placebo) | | | | | | | | | | |
| 5 | RCT | Serious^a^ | Not serious | Not serious | Not serious | None^c^ | 579/816 (70.9%) | 236/523 (45.1%) | OR 2.33 (2.08 to 2.62) | ⨁⨁⨁◯ MODERATE |
| Submental volume reduction ≥ 10% (ATX 2 mg/cm^2^ vs Placebo) | | | | | | | | | | |
| 2 | RCT | Serious^a^ | Not serious | Not serious | Serious^b^ | None^c^ | 222/514 (43.2%) | 26/508 (5.11%) | OR 8.43 (5.73 to 12.43) | ⨁⨁◯◯ LOW |
| Fibrosis (ATX 1 mg/cm^2^ vs. Placebo) | | | | | | | | | | |
| 2 | RCT | Serious^a^ | Not serious | Not serious | Serious^b^ | None^c^ | 40/237 (16.9%) | 4/236 (1.7%) | OR 10.04 (3.64 to 27.68) | ⨁⨁◯◯ LOW |
| Fibrosis (ATX 2 mg/cm^2^ vs. Placebo) | | | | | | | | | | |
| 5 | RCT | Serious^a^ | Not serious | Not serious | Not serious | None^c^ | 183/818 (22.4%) | 18/774 (2.3%) | OR 9.74 (6.08 to 15.61) | ⨁⨁⨁◯ MODERATE |
| Pain (ATX 1 mg/cm^2^ vs. Placebo) | | | | | | | | | | |
| 2 | RCT | Serious^a^ | Not serious | Not serious | Serious^b^ | None^c^ | 199/237 (84.0%) | 65/236 (27.5%) | OR 3.04 (2.46 to 3.77) | ⨁⨁◯◯ LOW |
| Pain (ATX 2 mg/cm^2^ vs. Placebo) | | | | | | | | | | |
| 5 | RCT | Serious^a^ | Serious^d^ | Not serious | Not serious | None^c^ | 603/818 (73.7%) | 236/774 (30.5%) | OR 2.38 (1.86 to 3.04) | ⨁⨁◯◯ LOW |
| Hematoma (ATX 1 mg/cm^2^ vs. Placebo) | | | | | | | | | | |
| 2 | RCT | Serious^a^ | Not serious | Not serious | Serious | None^c^ | 137/237 (57.8%) | 107/236 (45.3%) | OR 1.27 (1.07 to 1.52) | ⨁⨁◯◯ LOW |
| Hematoma (ATX 2 mg/cm^2^ vs. Placebo) | | | | | | | | | | |
| 5 | RCT | Serious^a^ | Not serious | Not serious | Not serious | None^c^ | 532/818 (65.0%) | 478/774 (61.8%) | OR 1.06 (0.98 to 1.14) | ⨁⨁⨁◯ MODERATE |
| Erythema (ATX 1 mg/cm^2^ vs. Placebo) | | | | | | | | | | |
| 2 | RCT | Serious^a^ | Not serious | Not serious | Serious^b^ | None^c^ | 96/237 (40.5%) | 43/236 (18.2%) | OR 1.80 (1.36 to 2.39) | ⨁⨁◯◯ LOW |
| Erythema (ATX 2 mg/cm^2^ vs. Placebo) | | | | | | | | | | |
| 5 | RCT | Serious^a^ | Not serious | Not serious | Not serious | None^c^ | 239/818 (29.2%) | 144/774 (18.6%) | OR 1.61 (1.35 to 1.93) | ⨁⨁⨁◯ MODERATE |
| Numbness (ATX 1 mg/cm^2^ vs. Placebo) | | | | | | | | | | |
| 2 | RCT | Serious^a^ | Not serious | Not serious | Serious^b^ | None^c^ | 109/237 (46.0%) | 5/236 (2.1%) | OR 21.78 (9.04 to 52.44) | ⨁⨁◯◯ LOW |
| Numbness (ATX 2 mg/cm^2^ vs. Placebo) | | | | | | | | | | |
| 5 | RCT | Serious^a^ | Not serious | Not serious | Not serious | None^c^ | 497/818 (60.8%) | 36/774 (25.1%) | OR 13.09 (9.48 to 18.08) | ⨁⨁⨁◯ MODERATE |
| Swelling (ATX 2 mg/cm^2^ vs. Placebo) | | | | | | | | | | |
| 3 | RCT | Serious^a^ | Not serious | Not serious | Not serious | None^c^ | 198/575 (34.4%) | 83/538 (15.4%) | OR 2.22 (1.76 to 2.79) | ⨁⨁⨁◯ MODERATE |
| Edema (ATX 2 mg/cm^2^ vs. Placebo) | | | | | | | | | | |
| 3 | RCT | Serious^a^ | Not serious | Not serious | Not serious | None^c^ | 337/575 (58.6%) | 155/538 (28.8%) | OR 2.06 (1.78 to 2.39) | ⨁⨁⨁◯ MODERATE |
| Pruritus (ATX 2 mg/cm^2^ vs. Placebo) | | | | | | | | | | |
| 3 | RCT | Serious^a^ | Not serious | Not serious | Serious^b^ | None^c^ | 71/575 (12.3%) | 33/538 (6.1%) | OR 2.00 (1.35 to 2.97) | ⨁⨁◯◯ LOW |
| Nodule (ATX 2 mg/cm^2^ vs. Placebo) | | | | | | | | | | |
| 3 | RCT | Serious^a^ | Not serious | Not serious | Serious^b^ | None^c^ | 77/575 (13.4%) | 13/538 (2.4%) | OR 5.66 (3.16 to 10.14) | ⨁⨁◯◯ LOW |
| Headache (ATX 2 mg/cm^2^ vs. Placebo) | | | | | | | | | | |
| 3 | RCT | Serious^a^ | Not serious | Not serious | Serious^b^ | None^c^ | 47/575 (8.2%) | 21/538 (3.9%) | OR 2.07 (1.26 to 3.41) | ⨁⨁◯◯ LOW |
| Paresthesia (ATX 2 mg/cm^2^ vs. Placebo) | | | | | | | | | | |
| 3 | RCT | Serious^a^ | Serious^d^ | Not serious | Serious^b^ | None^c^ | 70/575 (12.2%) | 19/538 (3.5%) | OR 3.25 (2.04 to 5.18) | ⨁◯◯◯ VERY LOW |
| Nasopharyngitis (ATX 2 mg/cm^2^ vs. Placebo) | | | | | | | | | | |
| 2 | RCT | Serious^a^ | Not serious | Not serious | Serious^b^ | None^c^ | 35/514 (6.8%) | 39/506 (7.7%) | OR 0.88 (0.57 to 1.36) | ⨁⨁◯◯ LOW |
| SLRS improvement or no change (ATX 1 mg/cm^2^ vs. Placebo) | | | | | | | | | | |
| 2 | RCT | Serious^a^ | Not serious | Not serious | Not serious | None^c^ | 216/237 (91.1%) | 216/236 (91.5%) | OR 1.00 (0.94 to 1.05) | ⨁⨁⨁◯ MODERATE |
| SLRS improvement or no change (ATX 2 mg/cm^2^ vs. Placebo) | | | | | | | | | | |
| 5 | RCT | Serious^a^ | Not serious | Not serious | Not serious | None^c^ | 761/818 (93.0%) | 709/774 (91.6%) | OR 1.01 (0.98 to 1.04) | ⨁⨁⨁◯ MODERATE |

CI, Confidence Interval; OR, Odds Ratio.

^a^ Risk of bias from conflicts of interest ‒ Reduced by one level.

^b^ Number of events lower than 400 ‒ Reduced by one level.

^c^ Publication bias not assessed.

^d^ Unexplained high statistical heterogeneity (I^2^ >50%) and/or no effect estimate overlap ‒ Reduced by one level.

Evidence levels of the GRADE workgroup.

High certainty: Strongly confident the true effect is close to the effect estimate.

Moderate certainty: Moderately confident in the effect estimate: The true effect might be close to the effect estimate, but it might be substantially different.

Low certainty: Limited confidence in the effect estimate: The true effect might substantially differ from the effect estimate.

Very low certainty: Little confidence in the effect estimate: The true effect will probably substantially differ from the effect estimate.
